# Effectiveness of Mass Media Campaigns to Reduce Alcohol Consumption and Harm: A Systematic Review

**DOI:** 10.1093/alcalc/agx094

**Published:** 2018-01-10

**Authors:** Ben Young, Sarah Lewis, Srinivasa Vittal Katikireddi, Linda Bauld, Martine Stead, Kathryn Angus, Mhairi Campbell, Shona Hilton, James Thomas, Kate Hinds, Adela Ashie, Tessa Langley

**Affiliations:** 1 Division of Epidemiology and Public Health, University of Nottingham, Nottingham, UK; 2 UK Centre for Tobacco & Alcohol Studies, Nottingham, UK; 3 MRC/CSO Social and Public Health Sciences Unit, University of Glasgow, Glasgow, UK; 4 Institute for Social Marketing, University of Stirling, Stirling, UK; 5 Institute of Education, University College London, London, UK

## Abstract

**Aims:**

To assess the effectiveness of mass media messages to reduce alcohol consumption and related harms using a systematic literature review.

**Methods:**

Eight databases were searched along with reference lists of eligible studies. Studies of any design in any country were included, provided that they evaluated a mass media intervention targeting alcohol consumption or related behavioural, social cognitive or clinical outcomes. Drink driving interventions and college campus campaigns were ineligible. Studies quality were assessed, data were extracted and a narrative synthesis conducted.

**Results:**

Searches produced 10,212 results and 24 studies were included in the review. Most campaigns used TV or radio in combination with other media channels were conducted in developed countries and were of weak quality. There was little evidence of reductions in alcohol consumption associated with exposure to campaigns based on 13 studies which measured consumption, although most did not state this as a specific aim of the campaign. There were some increases in treatment seeking and information seeking and mixed evidence of changes in intentions, motivation, beliefs and attitudes about alcohol. Campaigns were associated with increases in knowledge about alcohol consumption, especially where levels had initially been low. Recall of campaigns was high.

**Conclusion:**

Mass media health campaigns about alcohol are often recalled by individuals, have achieved changes in knowledge, attitudes and beliefs about alcohol but there is little evidence of reductions in alcohol consumption.

**Short summary:**

There is little evidence that mass media campaigns have reduced alcohol consumption although most did not state that they aimed to do so. Studies show recall of campaigns is high and that they can have an impact on knowledge, attitudes and beliefs about alcohol consumption.

## INTRODUCTION

Alcohol consumption is a major risk factor for adverse health, accounting for 2.3 million global deaths annually and representing the ninth greatest risk factor for disability-adjusted life-years (GBD 2015 Risk Factors Collaborators, 2016). In most countries, the trend in alcohol consumption is either increasing or stable (WHO, 2014), indicating a need for effective population-level strategies to reduce consumption and prevent related harms. Price increases and restrictions on the availability of alcohol can reduce alcohol-related harm (Anderson *et al.*, 2009; Martineau *et al.*, 2013; Allamani *et al.*, 2017).

Other population-level strategies include education and information, often using mass media with an aim to communicate messages cost-effectively to large numbers of people.

Mass media campaigns can directly or indirectly lead to health behaviour change in populations, but existing evidence varies depending on the type of behaviour being targeted (Wakefield *et al.*, 2010). For example, there is a substantial body of evidence assessing their role in reducing tobacco use (Bala *et al.*, 2013) and promoting physical activity (Abioye *et al.*, 2013). However, it is unclear whether mass media is an effective strategy to reduce alcohol consumption and related harm.

There is some evidence that mass media campaigns can, under certain conditions, reduce drink driving (Elder *et al.*, 2004; Jepson *et al.*, 2010) but little evidence that they have reduced alcohol-related road accidents or related injuries and deaths (Yadav and Kobayashi, 2015). A meta-analysis of media interventions to reduce youth substance use reported that messages addressing alcohol were associated with desired changes (single group pre-post) in consumption, attitudes and knowledge (Derzon and Lipsey, 2002). A meta-analysis of US mass media interventions reported a small effect on alcohol consumption based on four studies (Snyder *et al.*, 2004). Other systematic review evidence suggests social norm campaigns targeting college students are ineffective at preventing alcohol misuse (Foxcroft *et al.*, 2015) and provides mixed evidence of the effectiveness of school-based campaigns (Foxcroft and Tsertsvadze, 2011). Responsible drinking campaigns conducted by the alcohol industry are perceived as ambiguous by audiences and are ineffective at changing behaviour (Smith *et al.*, 2006).

Other than the topics already highlighted, evaluations of alcohol-related campaigns have not been synthesized in a way that can inform current policy. The aim of this study was to systematically review evidence for the effectiveness of mass media public health campaigns to reduce alcohol consumption and related harms.

## METHOD

The review protocol was submitted to the International Prospective Register of Systematic Reviews (PROSPERO) ref. CRD42017054999. The Preferred Reporting Items for Systematic reviews and Meta-Analyses (PRISMA) were followed.

### Inclusion criteria

Studies evaluating a mass media campaign aimed at reducing alcohol consumption (and its determinants) were eligible for inclusion. Mass media campaigns were defined as purposeful use of mass media channels to influence health behaviours and the individual level determinants of health behaviours. Mass media channels included television, radio, cinema, online broadcasting, newspapers and magazines, leaflets/booklets, direct mail, outdoor advertising, email and digital media. Studies had to have reported at least one of the following outcomes: alcohol consumption; alcohol-related social cognitive variables (e.g. knowledge, intentions, social norms); exposure outcomes (e.g. campaign awareness, exposure, understanding); alcohol-related harm; health service usage. Studies of multi-component interventions were eligible if they assessed the specific effects of a mass media component. Reports of primary research studies of any study design and conducted in any country, reported in English, were eligible for inclusion in the review. Exclusion criteria are listed in the Supplementary material (Supplementary Table S1).

### Search strategy

The following databases were searched from date of inception to July 2016: Medline, EMBASE, PubMed, Cochrane Library, Web of Science, SCOPUS, ASSIA and ERIC. The search terms used for Medline are shown in the Supplementary material (Supplementary Table S2) and were adapted for each database. Titles and abstracts were imported to an online database (Thomas *et al.*, 2010) and screened for relevance by one of a team of four reviewers. Full-text reports of all potentially eligible studies were retrieved and assessed for eligibility by one reviewer. A second reviewer assessed random samples of included (*n* = 10) and excluded (*n* = 10) studies at an early stage of the screening process to check agreement with the decisions and checked a further random sample (*n* = 20) once screening was complete. Conference abstracts of eligible studies were included only if a full-text paper of the same study could be located via searches of PubMed, Web of Science and Google Scholar. References of included studies were searched for any further potentially relevant studies.

### Data extraction

Study and campaign characteristics and relevant outcome data were extracted. Study design classifications were guided by the Cochrane Handbook tables of study design features (Reeves *et al.*, 2011). A second reviewer double-extracted data from a sample of studies and the two versions were checked for agreement. A further sample of studies was checked for accuracy by a second reviewer.

### Quality assessment

Included studies were assessed for methodological quality using the Effective Public Health Practice Project (EPHPP) Quality Assessment Tool for Quantitative Studies. Assessments were checked for accuracy by a second reviewer. The tool has six scored domains: selection bias, study design, confounders, blinding, data collection methods and withdrawal and dropouts. The overall quality of a study can be rated as strong, moderate or weak. Studies rated as weak on at least two domains are assigned an overall rating of weak.

### Synthesis

A narrative synthesis was conducted first to synthesize evidence of behaviour change and then by its determinants, including social cognitive and exposure outcomes. We privilege studies with high quality within the narrative synthesis (Katikireddi *et al.*, 2015). Due to study heterogeneity, a meta-analysis was not possible.

## RESULTS

### Study selection

**Fig. 1.**
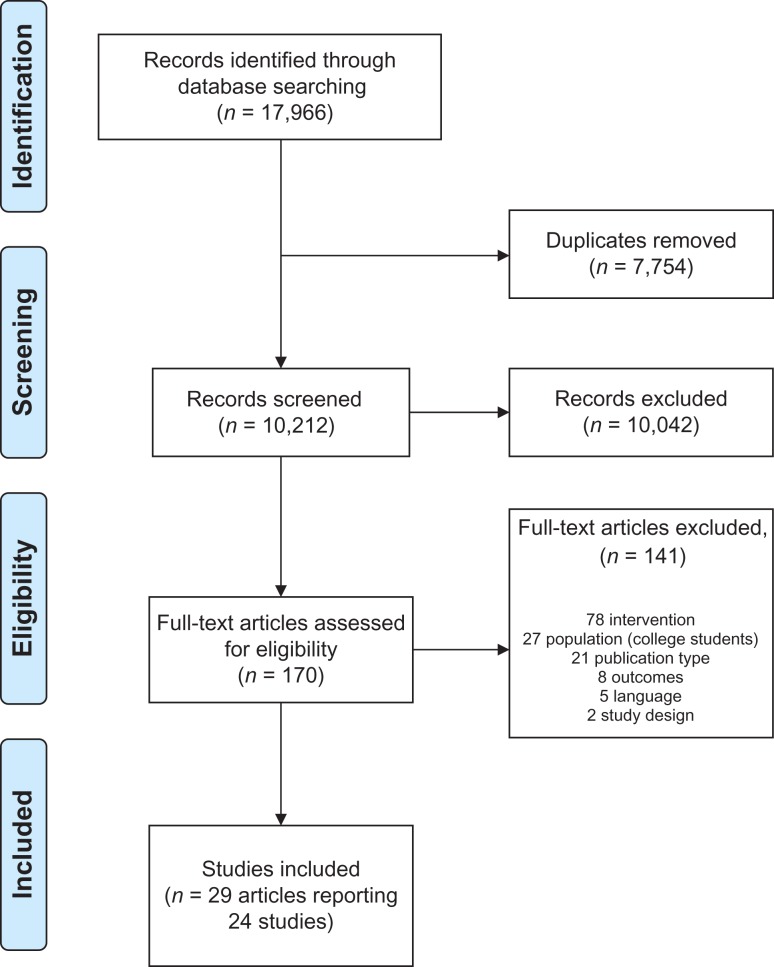
PRISMA flow diagram.

Searches produced 10,212 unique results and 170 of these were assessed for eligibility as a full-text report (Fig. 1). Twenty-nine papers were eligible for inclusion in the review, reporting 24 studies. Characteristics of included studies are shown in Table 1. Eight studies were conducted in the USA, five in Australia, two each in Finland, New Zealand and the UK, and one each in Canada, Denmark, Italy, the Netherlands and Sri Lanka. No campaigns were described as alcohol industry-funded.

**Table 1.** Characteristics of included studies

References and study design	Population	Campaign
Campaigns targeting general adult populations		
Allamani *et al.* (2000) Cross-sectional	*Campaign location and reach* Rifredi Health District (population 16,900), Florence, Italy. 5,000 carousels were disseminated *Campaign context* A component of a 6-year community alcohol project, which included a school program unit and training for healthcare workers and volunteers. The project had an aim to change local alcohol policy. Local TV and newspapers publicized the ‘carousel’ initiative prior to its implementation *Target population* Whole community *Comparison group* None	*Campaign objective* Increase awareness about responsible consumption of wine and other alcoholic drinks *Media channel(s)* Posters displayed in buses. ‘Carousel’ information tool (rotatable disk presented in a yellow envelope with ‘Take home a carousel’ printed on the outside) distributed via racks at GPs, pharmacies, schools, shops and bars, sent by mail to homes and distributed at local events
Barber *et al.* (1989) Cluster non-randomized controlled trial (exposure to pre-campaign letter in both groups was randomized at the individual level, forming a 2 × 2 design)	*Campaign location and reach* Townsville, North Queensland, Australia. Local reach *Target population* Adult alcohol drinkers *Comparison group* Community (Cairns) not exposed to TV advertisement but sent pre-campaign letters	*Campaign objective* Reduce alcohol consumption *Media channel(s)* TV advertisement, pre-campaign postal letter
Barber (1990) Uncontrolled before and after study with a separate exposed group measured post-campaign only	*Campaign location and reach* Australia, national reach *Campaign context* The beginning of a government 3-year National Campaign Against Drug Abuse *Target population* The general population *Comparison group* None.	*Campaign objective* Educate the public in the responsible use of drugs, with an emphasis on attitudes. Sought to raise public concern about the prevention of drug abuse generally *Media channel(s)* Radio, television and newspaper advertisements. Printed glossy booklet delivered to homes
Casswell *et al.* (1990) Cluster non-randomized controlled trial with separate repeated cross-sectional component	*Campaign location and reach* Four cities in New Zealand (each of 40,000–60,000 population). Local media channels used *Target population* Initially males 18–30 years, subsequently males 16–20 years. *Comparison group* Group exposed to mass media campaign plus community action. Control group not exposed to mass media or community action.	*Campaign objective* Increase awareness and support for relevant public policy on alcohol use. Change attitudes about alcohol use (more moderated drinking patterns and shift to non-alcoholic drinks). Wider community-level objectives included an increasing the amount of alcohol-related material (excluding industry promotion) in the local print media and radio programmes *Media channel(s)* Television, radio, newspaper, posters, cinema advertisements
Dixon *et al.* (2015) Interrupted time series	*Campaign location and reach* Western Australia, state-wide *Target population* Women aged 25–54 *Comparison group* None	*Campaign objective* Increase awareness of the link between alcohol and cancer among women. Specifically, the campaign aimed to increase awareness of long-term risky drinking, particularly in relation to alcohol-caused cancer *Media channel(s)* TV advertisements supported by print advertisements, community posters, web-based information and unpaid media strategies
Grønbæk *et al.* (2001) Interrupted time series	*Campaign location and reach* Denmark, national reach *Target population* Different target groups in different years e.g. people in their forties, heavy drinkers, whole population *Comparison group* None	*Campaign objective* Highlight alcohol consumption in order to promote interest in and understanding of alcohol prevention and treatment. Raise awareness and knowledge among adults of sensible levels of alcohol consumption. Reduce the consumption of alcohol in the whole of society in order to prevent alcohol-related injuries. The long-term objective of the annual campaigns was to bring about a reduction in the consumption of alcohol in Denmark *Media channel(s)* Television spots, information trailers and advertisements, booklet, newspaper advertisements, direct mail, outdoor media, alcohol unit counter tools
Kaariainen *et al.* (2008) Cross-sectional	*Campaign location and reach* Tampere, Finland. All households (200,000 population and 90,000 households) *Target population* General population *Comparison group* None	*Campaign objective* Promote a change in the culture of alcohol consumption and increase open discussion about alcohol *Media channel(s)* A pamphlet, designed for the campaign, delivered to homes
Karlsson *et al.* (2005) Cluster quasi-randomized controlled trial	*Campaign location and reach* Helsinki, Finland. Eight postal areas (86,400 households) *Target population* Males 30–49 years *Comparison group* Eight postal areas not receiving the pamphlet (40,900 households)	*Campaign objective* Support self-control of drinking *Media channel(s)* Information pamphlet, specially designed and delivered to homes, the size of a CD cover
Plant *et al.* (1979) Cohort study with independent samples pre- and post-test	Campaign location and reach Scotland, UK. Regional reach *Target population* Alcoholics and the general public Comparison group None	Campaign objective Persuade alcoholics to seek treatment and educate the public about alcoholism and agencies available to help problem drinkers. The possibility was also envisaged that the campaign might lead to a reduction in alcohol consumption by the general public, at least in the short term, although this was not a primary objective Media channel(s) TV films and newspaper advertisements
Siriwardhana *et al.* (2013) Cross-sectional for mass media outcomes but study included a cluster-randomized controlled design	*Campaign location and reach* Sri Lanka, rural village, local reach *Target population* Adult males *Comparison group* None for mass media outcomes	*Campaign objective* Educate the community about low-risk drinking (less than the equivalent of three standard drinks a day). Highlight the benefits of restricting amounts of drinking *Media channel(s)* Posters, recordings of street dramas distributed on DVD and leaflets delivered to homes
Wallack (1982) Repeated cross-sectional with control group	*Campaign location and reach* About half of Oakland and the city of San Leandro in Alameda County and the cities of El Cerrito, Richmond and San Pablo in Contra Costa County, California, USA *Target population* Initially males 18–35 years. Expanded to include females 25–40 years, Spanish heritage people and youth 14–17 years *Comparison group* City of Stockton in San Joaquin County, California, USA	*Campaign objective* Reduce the consumption of alcoholic beverages and lower the incidence of alcohol-related problems in the general population. Encourage more responsible drinking practices among current drinkers and thus obviate the need for treatment. Increase awareness and level of information about alcohol. Change attitudes regarding alcohol use *Media channel(s)* Television, radio, billboard displays, bus cards
*Campaigns targeting young people and/or their parents*		
Atkinson *et al.* (2011) Qualitative	*Campaign location and reach* UK. 943,644 views (unclear if all from UK) *Target population* Young people *Comparison group* None	*Campaign objective* Create peer to peer conversations regarding the negative effects of binge drinking. The long-term aim of the campaign was to create behaviour change by delivering sensible drinking messages in a non-patronizing way through the Hollyoaks brand *Media channel(s)* Online video reinforced and promoted through online discussion boards, character social media pages and blogs/video blogs, interviews with actors and interactive features such as a quiz, which assessed viewers’ recall of storylines, alcohol units and binge drinking knowledge and statistics relating to the negative effects of alcohol consumption
Flynn *et al.* (2006) Repeated cross-sectional with control group	*Campaign location and reach* Vermont, USA. Local reach *Target population* Adolescents in 8 school districts in grades 4–5 at start of intervention and grades 7–8 at end, parents of youth ages 9–13 and retail clerks *Comparison group* Adolescents in grades 7–8 in the 8 school districts which received no intervention	*Campaign objective* Reduce demand for alcohol among early adolescents by changing specific mediators of alcohol use and control alcohol supply. Parent objectives were to increase communication and limit alcohol supply. Retailer component focused on reducing access to alcohol by underage customers *Media channel(s)* Television (youth and parents), radio (parents) and video (retail clerks)
Kelley *et al.* (2000) Repeated cross-sectional	*Campaign location and reach* Colorado, New Jersey and Washington, USA. Four rural communities each with populations of <30,000 *Target population* Adolescent females *Comparison group* None	*Campaign objective* Combat alcohol and tobacco use *Media channel(s)* Broadcast media (radio and TV), print media (newspaper, billboard), posters and tray liners in the school cafeteria and local fast food restaurants
Kypri *et al.* (2005) Cluster non-randomized controlled trial	*Campaign location and reach* Two communities in the south island of New Zealand. Districts of Ashburton (population 25,446) and Waitaki (20,088) *Target population* Parents of adolescents *Comparison group* Clutha district (population 17,172). All year 11, 12 and 13 students and from all households in the district with a teenager in years 9–13 at either of Clutha district’s secondary schools	*Campaign objective* (i) Increase the knowledge of adults in the Ashburton and Waitaki districts of the risks of supplying alcohol to teenagers; (ii) encourage a change of attitude such that a teenager’s parent is considered the only appropriate supplier of alcohol, and that teenage drinking should occur only under adult supervision; and (iii) effect a reduction in the percentage of adults who supply alcohol to teenagers for unsupervised consumption *Media channel(s)* Local newspaper, print media, local radio, media events, billboard advertisements, the distribution of printed material and the presentation of campaign information at point of sale. In two communities, a range of awareness-raising events for youth and adults were held
Scheier (2010) Age-cohort study	*Campaign location and reach* USA, national campaign *Target population* Youth 9–18 years and their parents. Other influential adults (e.g. staff at alcohol selling outlets) *Comparison group* None	*Campaign objective* Educate and enable America’s youths to reject illegal drugs. Reduce adolescent initiation of drug use. Curtail use among those already engaged *Media channel(s)* Radio, television, newsprint, magazines, movies, billboards, advertisements on buses, at malls, at sports events
Surkan *et al.* (2003) Cross-sectional	*Campaign location and reach* Massachusetts, USA. Radio stations reaching Boston, Worcester, Cape Cod, Franklin County, New Bedford area and Springfield area *Target population* Parents *Comparison group* None	*Campaign objective* Promote parent–child communication about alcohol use *Media channel(s)* Radio advertisement (paid)
Trees (2015) Cross-sectional and qualitative	*Campaign location and reach* Broome and surrounding areas, Western Australia. Local reach *Target population* Indigenous youth in Broome and the wider Kimberley region (the broadcast area of Goolarri TV and Radio) *Comparison group* None	*Campaign objective* Alcohol awareness *Media channel(s)* Television and radio (both local)
van Gemert *et al.* (2011) Cross-sectional	*Campaign location and reach* Australia, national reach *Target population* Young people 15–25 years and their parents *Comparison group* None	*Campaign objective* Raise awareness of the harms and costs associated with risky drinking among young Australians, and to deliver personally relevant messages to encourage, motivate and support the primary target groups to modify their behaviour *Media channel(s)* A range of mass media strategies and outlets including television, cinema, radio, online advertising, brochures and out-of-home print advertisements such as free postcard advertising, washroom mirrors in nightclubs, street posters, stencil chalking and on street furniture
van Leeuwen (2013) Cohort study	*Campaign location and reach* Netherlands, national reach *Target population* Less educated adolescents (high school students receiving preparatory middle-level applied education) *Comparison group* Participants who reported that they had seen one episode or less and did not complete any of the five surveys between pre- and post-test	*Campaign objective* Favourably influence beliefs about the consequences of substance use, e.g. as being damaging to health, intentions, and behaviour concerning the use of substances *Media channel(s)* National TV and online viewing via an emailed link
*Campaigns targeting pregnant women or women of childbearing age*		
Awopetu *et al.* (2008) Historically controlled study	*Campaign location and reach* Essex and Atlantic Counties, New Jersey, USA. Unknown reach as messages were communicated along major transit routes *Target population* Women of childbearing age *Comparison group* None but the authors narratively compared the outcome to that achieved in a historic period	*Campaign objective* Urge women to not drink alcohol if they are pregnant and to avoid alcohol if they could become pregnant in order to reduce risks *Media channel(s)* Billboard posters along transit routes, interiors of subway trains and city buses, local newspapers, radio public service announcements, printed materials (countertop inserts and brochures)
Casiro *et al.* (1994) Interrupted time series	*Campaign location and reach* Manitoba, Canada. Province-wide campaign *Target population* General public and all physicians in Manitoba *Comparison group* None	*Campaign objective* Increase awareness of the dangers of drinking during pregnancy *Media channel(s)* TV, brochure
Hanson *et al.* (2012) Cross-sectional	*Campaign location and reach* American Indian communities in the Northern Plains, USA *Target population* General population *Comparison group* None	*Campaign objective* Increase awareness of foetal alcohol syndrome disease, the effects of alcohol on the unborn child and reduce alcohol consumption *Media channel(s)* Posters, radio adverts, newspaper adverts, brochures
Lowe *et al.* (2010) Cluster quasi-randomized controlled trial	*Campaign location and reach* Iowa, USA. Ten agencies/sites *Target population* Pregnant women *Comparison group* Women in 10 agencies randomized to receive advice and opportunity to watch TV commercial only (not exposed to the videotape/DVD+pamphlet)	*Campaign objective* Increase interpersonal discussions and knowledge about the dangers of alcohol use during pregnancy *Media channel(s)* Videotape/DVD, printed pamphlet (both groups exposed to TV commercial— no details reported)

### Study quality

Two studies were rated strong quality (Flynn *et al.*, 2006; Scheier and Grenard, 2010), four were rated moderate quality (Wallack and Barrows, 1982; Barber and Grichting, 1990; Kypri *et al.*, 2005; Lowe *et al.*, 2010;) and 18 were rated weak quality (Plant *et al.*, 1979; Barber *et al.*, 1989; Casswell *et al.*, 1990; Casiro *et al.*, 1994; Allamani *et al.*, 2000; Kelley *et al.*, 2000; Grønbæk *et al.*, 2001; Surkan *et al.*, 2003; Karlsson *et al.*, 2005; Awopetu *et al.*, 2008; Kaariainen *et al.*, 2008; Atkinson *et al.*, 2011; van Gemert *et al.*, 2011; Hanson *et al.*, 2012; Siriwardhana *et al.*, 2013; van Leeuwen *et al.*, 2013; Dixon *et al.*, 2015; Trees, 2015). EPHPP tool domain ratings indicated 20 studies did not report reliability and validity of data collection tools, ten studies had high risk of selection bias and nine were rated weak on study design (Table 2).

**Table 2.** EPHPP quality assessment ratings

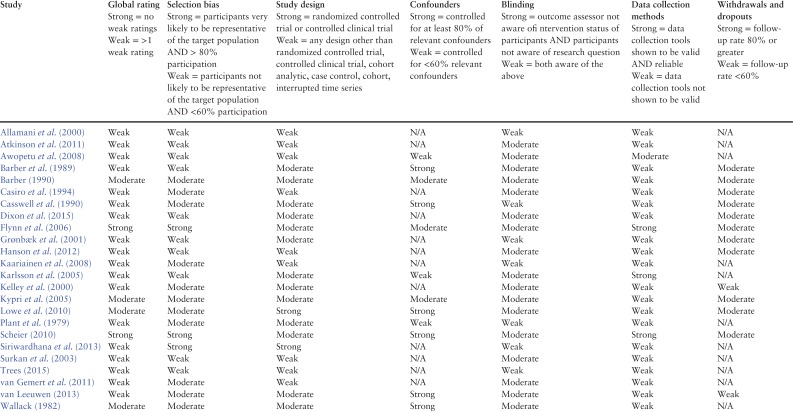							

### Synthesis of results

Table 3 summarizes the findings of included studies, structured by different types of outcomes: health, social and behavioural outcomes (e.g. mortality, societal change, health behaviour), health promotion outcomes (e.g. knowledge, attitudes, behavioural intentions) and exposure outcomes (e.g. recall, understanding, onward transmission). More detailed results of included studies are shown in the Supplementary Table S3.

**Table 3.** Results of included studies

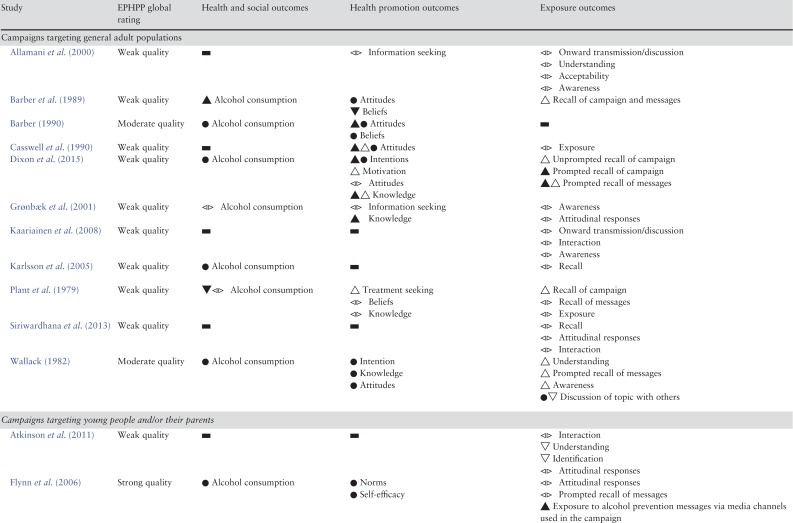							
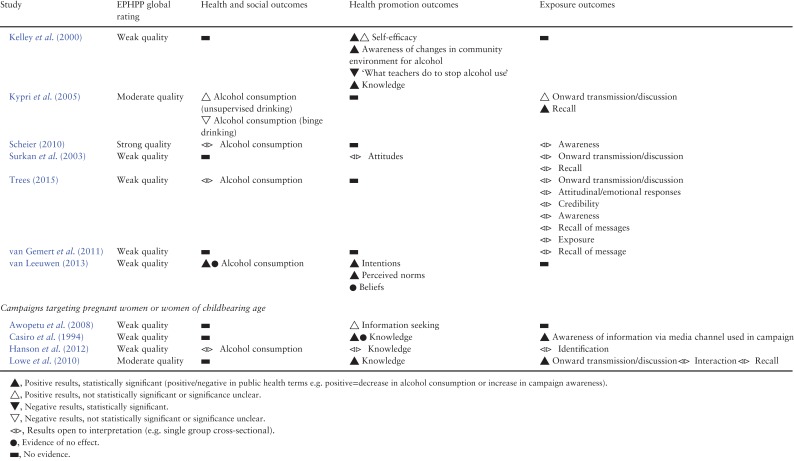							

### Alcohol consumption

Thirteen studies reported the effects of mass media campaigns on alcohol consumption. Six of the campaigns aimed to reduce consumption (Wallack and Barrows, 1982; Barber *et al.*, 1989; Grønbæk *et al.*, 2001; Karlsson *et al.*, 2005; Flynn *et al.*, 2006; Scheier and Grenard, 2010) while the other seven aimed only to impact knowledge (Plant *et al.*, 1979; Barber and Grichting, 1990; Kypri *et al.*, 2005; Hanson *et al.*, 2012; Dixon *et al.*, 2015; Trees, 2015), beliefs (van Leeuwen *et al.*, 2013), attitudes (Kypri *et al.*, 2005), treatment seeking (Plant *et al.*, 1979) or supply of alcohol (Kypri *et al.*, 2005). There was little evidence of reductions in alcohol consumption associated with exposure to campaigns. Six of the studies compared exposed and non-exposed groups, or exposed groups over time, five reporting no statistically significant differences in alcohol consumption (1 strong quality, 3 moderate, 1 weak) (Wallack and Barrows, 1982; Barber and Grichting, 1990; Karlsson *et al.*, 2005; Kypri *et al.*, 2005; Flynn *et al.*, 2006). One study (weak quality) found a significant effect of a TV and mailed letter campaign (Barber *et al.*, 1989). Consumption on a typical day decreased 47%, contrasting with increases in groups receiving either the TV or letter components or no intervention. Of four studies that examined associations between campaign viewing or awareness (rather than group allocation) and alcohol consumption, one study (strong quality) reported that increases in campaign awareness in older adolescence, but not younger adolescence, was associated with decreases in binge drinking (Scheier and Grenard, 2010), one study (weak quality) reported campaign viewing status was a significant predictor of number of drinks consumed per occasion (van Leeuwen *et al.*, 2013) and two studies of weak quality found no significant difference in consumption (Plant *et al.*, 1979; Dixon *et al.*, 2015).

### Treatment/information seeking

There was some evidence that mass media campaigns generated increases in treatment seeking or information seeking, from a total of four studies reporting this outcome (all weak quality). One of the campaigns had an aim to promote interest in and understanding of alcohol treatment (Grønbæk *et al.*, 2001) while three campaigns had other aims (Plant *et al.*, 1979; Allamani *et al.*, 2000; Awopetu *et al.*, 2008;). New referrals for alcoholism increased by 65% following a TV and radio campaign (Plant *et al.*, 1979). Forty-nine Foetal Alcohol Syndrome-related telephone calls were received by a Family Health Line following a campaign, compared to 5–6 calls received in a historical period (Awopetu *et al.*, 2008). Evaluation of a long-term national annual campaign found 6–7% had obtained an alcohol unit counter and 2% (~80,000 people) had used or considered using it (Grønbæk *et al.*, 2001). The other study reported mixed qualitative evidence which was difficult to interpret (Allamani *et al.*, 2000).

### Intentions and motivation

Three studies reported intentions to reduce alcohol consumption. One of the campaigns aimed to reduce consumption (Wallack and Barrows, 1982), one aimed to influence beliefs (van Leeuwen *et al.*, 2013) and one aimed to promote knowledge (Dixon *et al.*, 2015). The first study (moderate quality) reported that some respondents indicated they might change their behaviour but no further data were provided (Wallack and Barrows, 1982). The second study (weak quality) compared those who reported they had seen the campaign to those who did not. Viewing status significantly predicted changes in intentions to decrease alcohol use; viewers increased their intentions whereas non-viewers decreased their intentions to reduce alcohol use (van Leeuwen *et al.*, 2013). In the third study (weak quality), the proportion who responded that they were likely to reduce their alcohol consumption increased significantly from 17 pre-test to 30% post-test. However, there was no difference in intentions to reduce consumption when comparing drinkers who were aware and not aware of the campaign (Dixon *et al.*, 2015). In the single study that measured motivation to reduce alcohol consumption, approximately half those who drank alcohol and recognized the campaign reported that it made them feel motivated (either very or somewhat) to reduce their alcohol consumption (Dixon *et al.*, 2015).

### Beliefs and attitudes

Five studies measured alcohol-related beliefs or attitudes, some observing changes in the desired direction. Two of the campaigns aimed to change beliefs or attitudes (Barber and Grichting, 1990; Casswell *et al.*, 1990), two aimed to reduce consumption (Wallack and Barrows, 1982; Barber *et al.*, 1989) and one aimed to promote treatment seeking and improve knowledge (Plant *et al.*, 1979). A national campaign targeting a range of drugs reported a statistically significant increase in support for higher tax on alcohol and for banning alcohol in public places (moderate quality) (Barber and Grichting, 1990). However, there was no significant change pre- and post-campaign in the proportions who consider alcohol to be a drug, the perceived danger associated with alcohol or in support for a range of other policies aimed at limiting consumption. A study (moderate quality) of a campaign involving television, radio, billboard displays and bus cards reported that respondents remained consistent over time in their concern about how much alcohol they consume and the possible negative effects (Wallack and Barrows, 1982). Other findings were from studies of weak quality and produced mixed findings on a number of beliefs and attitudes (Plant *et al.*, 1979; Barber *et al.*, 1989; Casswell *et al.*, 1990).

### Knowledge

Eight studies reported the impact of mass media campaigns on alcohol-related knowledge, with evidence that knowledge can be increased. Seven of the campaigns aimed to promote knowledge (Plant *et al.*, 1979; Wallack and Barrows, 1982; Casiro *et al.*, 1994; Grønbæk *et al.*, 2001; Lowe *et al.*, 2010; Hanson *et al.*, 2012; Dixon *et al.*, 2015;) while one aimed to reduce consumption (Kelley *et al.*, 2000). Of two studies of moderate quality, one found a significant improvement in knowledge of the risks of alcohol use during pregnancy in an exposed group compared to a control group (Lowe *et al.*, 2010). The other study described no changes in knowledge in youth and adult samples during a campaign, but participants were already well informed at baseline; nevertheless slightly more than 20% of youth indicated they had received new information as a result of the campaign (Wallack and Barrows, 1982). The remaining six studies were of weak quality. One found a significant improvement in knowledge that drinking alcohol on a regular basis increases cancer risk and of the recommended number of standard drinks for low-risk in the long-term (Dixon *et al.*, 2015). A repeated annual campaign reported an immediate increase in knowledge of unit guidelines after each campaign with a steady increase over time (Grønbæk *et al.*, 2001). One study reported a significantly higher proportion of respondents after the campaign knew that alcohol will reach the baby in a pregnant woman, and that drinking alcohol during pregnancy could cause mental, physical and behavioural abnormalities in the baby. There was also a significant increase in knowledge of risk to the baby of drinking small amounts of alcohol (drinking once a week or once a month) but not of more regular drinking (once a day). Knowledge levels did not significantly change on other statements (Casiro *et al.*, 1994). One study found high proportions of participants agreed the campaign increased their knowledge on foetal alcohol syndrome and on the effect of alcohol consumption during pregnancy (Hanson *et al.*, 2012). One study reported a significant increase in how much had been learned from the media about the dangers of alcohol use (Kelley *et al.*, 2000). Finally, those who reported being exposed to a campaign demonstrated slightly improved ability to name people or agencies offering help to problem drinkers and to name symptoms of alcoholism (Plant *et al.*, 1979).

### Other social cognitive outcomes

Two studies reported self-efficacy to reduce or stop the consumption of alcohol; one found no effect on self-efficacy (strong quality) (Flynn *et al.*, 2006) and the other found that increases in self-efficacy year-on-year were either statistically significant or of borderline significance (weak quality) (Kelley *et al.*, 2000). A single study reporting perceived social norms found that viewing the campaign was associated with an increase in perceived social pressure to limit consumption (weak quality) (van Leeuwen *et al.*, 2013).

### Exposure outcomes

#### Interaction, discussion or onward transmission

Evidence that campaigns promoted interaction or discussion about alcohol was mixed and mostly weak. More individuals exposed to a campaign had talked to friends about alcohol use during pregnancy compared to controls. The difference was of borderline significance and the campaign aimed to promote interpersonal discussion about the topic (moderate quality) (Lowe *et al.*, 2010). A campaign which had an objective of reducing parental supply of alcohol to adolescents reported that 28% of parents in the media areas said they discussed issues surrounding unsupervised drinking more with their teenager during the campaign than before it commenced, of whom 76% attributed this to the campaign, while 20% said they discussed unsupervised drinking more frequently with other adults (moderate quality) (Kypri *et al.*, 2005). Three other studies were of weak quality and their designs did not allow assessment of causal associations (Surkan *et al.*, 2003; Atkinson *et al.*, 2011; Siriwardhana *et al.*, 2013;).

#### Recall

Seventeen studies reported participant recall, recognition or awareness of mass media campaigns (2 strong quality, 3 moderate and 12 weak) (Plant *et al.*, 1979; Wallack and Barrows, 1982; Barber *et al.*, 1989; Casiro *et al.*, 1994; Allamani *et al.*, 2000; Grønbæk *et al.*, 2001; Surkan *et al.*, 2003; Karlsson *et al.*, 2005; Kypri *et al.*, 2005; Flynn et al., 2006; Kaariainen *et al.*, 2008; Lowe *et al.*, 2010; Scheier and Grenard, 2010; van Gemert et al., 2011; Siriwardhana *et al.*, 2013; Dixon *et al.*, 2015; Trees, 2015). One study compared unprompted recall in an exposed and a non-exposed group, finding levels of recall in the groups were 65 and 9%, respectively (Barber *et al.*, 1989). Based on 12 of the 17 studies, unprompted recall in exposed groups ranged from 5.7% in a local bus poster campaign (Allamani *et al.*, 2000) to 80% in a repeated national campaign (Grønbæk *et al.*, 2001). Four studies measured prompted recall of campaigns or campaign messages. The first study found 76% of the exposed group and 39% of the non-exposed group said they had seen at least one of the campaign advertisements (Wallack and Barrows, 1982). The proportion that had seen or heard at least one of the campaign messages was 81.3% in the second study (Flynn *et al.*, 2006). The third study found significantly more campaign items were reported as seen by an exposed group than a control group (Kypri et al., 2005) and the fourth study found 81.2% recalled the campaign advertisement after being shown it (Dixon *et al.*, 2015). Unprompted recall of campaign messages ranged from 12 to 96% based on six studies (Plant *et al.*, 1979; Barber *et al.*, 1989; Surkan *et al.*, 2003; van Gemert *et al.*, 2011; Dixon *et al.*, 2015; Trees, 2015).

#### Attitudinal/emotional responses

Six studies recorded attitudinal or emotional responses to mass media campaigns with generally positive results. For example, in a study of strong quality the proportions who liked the messages, of those who had seen or heard them, were 70 and 75%, respectively, for TV and radio (Flynn *et al.*, 2006). The proportion who thought a national campaign was a good or very good initiative was ~90% (weak quality) (Grønbæk *et al.*, 2001).

### Campaigns targeting specific population groups

Eleven campaigns targeted general adult populations, three of which targeted men (Casswell *et al.*, 1990; Karlsson *et al.*, 2005; Siriwardhana *et al.*, 2013) and one targeted women (Dixon *et al.*, 2015) (Table 1). Studies (mostly weak quality) suggest such adult-targeted campaigns can be recalled by the target audience and can achieve changes in knowledge, attitudes and beliefs about alcohol, but there is a lack of evidence that they can impact alcohol consumption. Nine campaigns targeted alcohol consumption in young people (Kelley *et al.*, 2000; Surkan *et al.*, 2003; Kypri *et al.*, 2005; Flynn *et al.*, 2006; Scheier and Grenard, 2010; Atkinson *et al.*, 2011; van Gemert *et al.*, 2011; van Leeuwen *et al.*, 2013; Trees, 2015) (Table 1). They utilized different strategies and provided mixed findings, some of which indicated they were effective in reaching their target audience and achieving their objectives but several of the studies were of very weak design. Four campaigns aimed to reduce alcohol consumption in pregnancy (Casiro *et al.*, 1994; Awopetu *et al.*, 2008; Lowe *et al.*, 2010; Hanson *et al.*, 2012). As with those targeting general adult populations, they provide evidence that they can be effective at improving knowledge and awareness in the target audience but the quality of the evidence is low.

## DISCUSSION

The evidence suggests mass media health campaigns about alcohol can be recalled by individuals and can achieve changes in knowledge, attitudes and beliefs about alcohol, based mainly on weak quality studies. Findings of studies that measured alcohol consumption suggest campaigns have not reduced consumption, although most did not state that they directly aim to do so.

The finding that campaigns can be recalled suggests appropriate media channels, targeting strategies, durations and intensities have been utilized to reach target audiences. These campaign characteristics were not always reported by studies so it is not possible to draw a link between types of campaign strategies and levels of recall or exposure. Recall of tobacco mass media campaigns has been shown to be positively associated with smoking cessation (Jepson *et al.*, 2007) so the outcome may be an important first step towards subsequent behaviour change in populations.

Most campaigns that aimed to improve knowledge were shown to be effective. This was particularly evident in areas where knowledge was initially low, for example, knowledge of unit consumption guidelines and of the link between alcohol and cancer. Mass media can yield sustained knowledge, which may lay the groundwork for reductions in consumption that are achieved using other public health measures.

There was evidence of increases in information seeking and treatment seeking. However, alcohol campaigns have not presented the simple call to action of tobacco messages (‘quit’) or provided offers of tangible help such as ‘quitlines’. Furthermore, as alcohol support services have historically been aimed at very heavy drinkers there may be a perception that current services do not cater for those who drink less. Mass media might therefore have limited utility in promoting service uptake.

Most studies found no impact on alcohol consumption, consistent with the conclusion of a previous review that there should be modest expectations of behaviour change from such campaigns (Snyder *et al.*, 2004). Longer term evaluations conducted following sustained and repeated exposure to campaigns might be expected to be better able to detect effects on behaviour. However, the relationship between tobacco mass media campaign duration and effectiveness has been difficult to gauge due to confounding influences and trends over time (Durkin *et al.*, 2012). The context in which alcohol health promotion campaigns operate is particularly challenging because of the ubiquity and power of alcohol marketing (de Bruijn *et al.*, 2016) and pro-alcohol cultural norms (Gordon *et al.*, 2012). This is another key difference to tobacco, where health campaigns in recent years have run in a context where most tobacco marketing has been banned or strictly regulated and social norms have become increasingly anti-smoking. The current review found evidence of impact on short term intermediate outcomes, suggesting mass media can play a supportive role for other actions which are more likely to have an impact on behaviour. These might include price-based measures (Babor *et al.*, 2010), advertising restrictions (Siegfried *et al.*, 2014), limiting availability and access to alcohol (Anderson *et al.*, 2009) with the targeting of high risk groups (Foxcroft *et al.*, 2015).

This review has the following strengths and limitations. It is the first comprehensive systematic review of evidence of the effectiveness of mass media to reduce alcohol consumption, allowing those who make decisions about whether and how to develop and implement such campaigns to do so informed by a synthesis of the evidence base. A strength of the review lies in the common features shared by all the included mass media campaigns as a result of focused inclusion criteria, such as incidental exposure and the absence of person-to-person contact. In addition to exploring effects of campaigns by outcome, the presentation of findings by common target population (general adults/young people/pregnant women) further strengthens the ability of the review to guide policy and practice. The review has also identified gaps in knowledge for further research. The quality of studies included in the review was generally weak, most outcomes were self-reported and evidence in high risk sub-groups was not reported consistently enough to be synthesized in the review. There is a need for evaluations of higher quality that demonstrate valid and reliable measurement of outcomes, adopt a cluster-randomized or robust natural experiment design where feasible and identify effects in high risk sub-groups. Aims of campaigns were extracted from included reports and were often limited in detail. For a better assessment of whether mass media campaigns achieve their aims, pre-campaign documents should be sought that set out *a priori* aims, against which study findings can be assessed, although such documents are unlikely to be available to researchers. The findings have limited generalizability beyond developed countries. The inclusion only of studies published in English and indexed in electronic databases may have introduced language and publication bias. Some older campaigns were conducted in a different media landscape to the current digital and online environment. However, the evidence was predominantly from campaigns involving TV and radio which are media channels that still have important influence today.

There are barriers to the conduct of evaluations of population-level interventions to the standards required to achieve a ‘strong’ quality rating. For example, it is usually not appropriate or feasible to conduct randomized controlled evaluations of such interventions. Similarly, high study response rates can be difficult to achieve in large-scale studies. When assessing participant attrition the tool does not take into account the length of follow-up, which could bias against longer term follow-ups. However, the EPHPP quality assessment tool allowed important core domains to be assessed and the quality of the evidence to be compared with other public health interventions. The use of the EPHPP tool within this review allowed studies of all designs and appropriate study domains to be assessed.

The review identified only 24 mass media alcohol campaigns, using searches without a time restriction, compared to 72 English-language alcohol harm reduction campaigns produced between 2006 and 2014 identified by a content analysis study (Dunstone *et al.*, 2017). Our synthesis of the evidence includes only the minority of campaigns that have been both evaluated and published.

To address the challenges in evaluating mass media alcohol campaigns, more studies are required of larger campaigns exploiting indirect as well as direct pathways to behaviour change. Campaign cost-effectiveness should also be assessed to establish whether any health benefits observed are sufficient to justify the substantial expenditure involved in campaign development and broadcast.

## CONCLUSION

Mass media health campaigns about alcohol are often recalled by individuals, have achieved changes in knowledge, attitudes and beliefs about alcohol but there is little evidence of impact on alcohol consumption. Such interventions may have a longer term role as part of a comprehensive harm reduction strategy, by improving knowledge in areas where it is low, potentially contributing to changing harmful drinking norms and helping to set the agenda for alcohol policy change.

## Supplementary Material

Supplementary Data
