# Supplementary material for: Effectiveness of Mass Media Campaigns to Reduce Alcohol Consumption and Harm: A Systematic Review
Source: Alcohol Alcohol. 2018 Jan 10;53(3):302–16. doi: 10.1093/alcalc/agx094 (PMC5913684; doi:10.1093/alcalc/agx094)
Supplement: Supplementary Data [file 20171011supportinginformation.docx]

Table S1. Exclusion criteria

| Studies of campaigns with the following characteristics were excluded:   - Involved individual person-to-person delivery - Required active engagement before receipt of the message rather than passive message exposure - Used a baseline measure to tailor or target a subsequent mass media message - Targeted drink-driving - Promoted alcohol consumption - Based in schools - Targeted college students within university campuses - Social norms campaigns where it was not evident from the information available that a mass media channel was used   The following types of studies were excluded:   - Campaign message testing - Evaluations of alcohol advertisement bans - Evaluations of warning labels on alcohol products - Evaluations of online treatment or self-help programmes |
| --- |

Table S2. Medline search terms

| \| 1 \| mass media.ti,ab. \| \| --- \| --- \| \| 2 \| mass communication.ti,ab. \| \| 3 \| social marketing.ti,ab. \| \| 4 \| broadcast*.ti,ab. \| \| 5 \| exp mass media/ \| \| 6 \| pamphlets/ \| \| 7 \| exp serial publications/ \| \| 8 \| electronic mail/ \| \| 9 \| social media/ \| \| 10 \| advert*.mp. \| \| 11 \| campaign*.mp. \| \| 12 \| or/1-11 \| \| 13 \| alcohol*.mp. \| \| 14 \| binge drink*.mp. \| \| 15 \| episodic drink*.mp. \| \| 16 \| or/13-15 \| \| 17 \| 12 and 16 \| \| 18 \| limit 17 to humans \| |
| --- | --- | --- | --- | --- | --- | --- | --- | --- | --- | --- | --- | --- | --- | --- | --- | --- | --- | --- | --- | --- | --- | --- | --- | --- | --- | --- | --- | --- | --- | --- | --- | --- | --- | --- | --- | --- |

Table S3. Results of included studies

| **Study**  **EPHPP rating** | **Intermediate/Distal/Process outcomes** | **Proximal outcomes** | **Media outcomes** | **Author conclusion** |
| --- | --- | --- | --- | --- |
| **Campaigns targeting general adult populations** | | | | |
| **Allamani (2000)**  Weak quality | None. | **Information seeking**  In one of the two pharmacies, clients seemed to be embarrassed about asking for further information on the carousel. In a middle school not only were the students interested in the carousels, but the custodians and some teachers not involved in the community project asked for further information. | **Onward transmission**  Carousel seen by at least one other person = 85.7% parents.  **Understanding**  Carousel tool easily understandable = 83% Co-op members (n = 129); 94% parents (n = 56).  **Acceptability**  Useful for increasing one’s information about the use of alcohol = 87% Co-op members; 89% parents.  Useful for helping to reflect on one’s own use of alcohol = 88% Co-op members; 83% parents.  Informative regarding local health services = 83% Co-op members; 69% parents.  Felt singled out as ‘alcohol abusers’ = 9% Co-op members.  Difficult to handle the disk = 3% Co-op members.  **Awareness**  Noticed the posters = 5.7% bus passengers (n = 210); 7% of sub-group of local residents (n = 115).  Informed about the Florence Community Project and familiar with some of its initiatives = 14.8% bus passengers; 27% of sub-group of local residents. | The visibility of the posters was low. The acceptability of the carousel was high. |
| **Barber (1989)**  Weak quality | **Alcohol consumption**  *Advertisement + letter* group reported significantly lower post-campaign alcohol consumption than the other three groups, measured as consumption yesterday, F(1, 88) = 4.03, p < 0.05, and consumption on a typical day, F(1, 87) = 4.15, p < 0.05. No significant effect of TV advertisement on alcohol consumption. Marginally significant effect of letter on alcohol consumption, F(4, 91) = 2.45, p = 0.05. No significant effect of the letter on typical weekend consumption in the exposed group. Significantly lower consumption in those sent a letter in the comparison group compared to those not sent a letter in the comparison group, F(1, 44) = 6.77, p < 0.05. | **Attitudes**  No significant effect of either TV advertisement or letter on any attitudes to alcohol measured. No significant differences between any of the four groups. Attitudes measured were: increase alcohol tax; favour compulsory treatment for alcoholics; keep underage drinking illegal; ban alcohol advertising; reduce alcohol tax; ban drinking in public buildings; introduce random breath testing; think moderate drinking is acceptable.  **Beliefs**  *Advertisement + letter* group had a lower perception of danger associated with alcohol consumption, F(1, 93) = 4.00, p < 0.05. No significant effect of either TV advertisement or letter on danger of alcohol ratings. | **Recall**  Of those who remembered seeing the advertisement, able to name at least one of the controlled-drinking behaviours promoted by the advertisement = 94% exposed group (95% letter; 93% no-letter); 0% comparison group. | A very high proportion of those who remembered seeing the advertisement were able to recall one of the campaign messages 3 weeks after the campaign. Both advertisement and pre-campaign letter may be required to influence short-term behaviour change. |
| **Barber (1990)**  Moderate quality | **Alcohol consumption**  Consumption was lower in both samples post-campaign than in the pre-campaign sample (non-significant difference).  Mean intake pure ethyl alcohol on previous day and previous weekend:  *Pre- and post-campaign sample*  Before = 42.0g  After = 38.9g  *Post-campaign only sample*  After = 36.6g | **Attitudes**  Repeated measures pre and post-campaign comparison:  Statistically significant increase in support for increasing tax on alcohol and decrease in support for reducing tax on alcohol. No statistically significant change in support for: Compulsory treatment for alcoholics; Selling alcohol to minors is rightly illegal; Ban alcohol advertisements; Ban alcohol in public places; Introduce random breath tests; Nothing wrong with alcohol in moderation.  Independent samples pre- and post-campaign comparison:  Increase in support for banning alcohol in public places, p < 0.001.  **Beliefs**  Consider alcohol to be a drug: no change pre- and post-campaign.  Perceived danger associated with alcohol: no statistically significant change pre- and post-campaign. | None. | There was a weak short-term effect on attitudes toward becoming more in favour of legislation aimed at limiting alcohol consumption. The effect was considerably enhanced by the pretesting procedure. |
| **Casswell (1990)**  Weak quality | None. | **Attitudes**  A small but statistically significant effect of treatment on each of the four policy variables. In three policy areas—advertising, general restrictions on alcohol availability and price—the overall treatment effect was due to a decline in support for alcohol policies in the non-exposed cities, while no significant changes occurred in the media cities. Non-significant increase over time in the media cities in reported intensity of opposition to liberalisation in respect of alcohol sales in supermarkets and age limits.  Personal controls on drinking (such as counting drinks and doing something energetic to unwind): significant shift towards less control over time within the media and non-exposed groups.  "Pro-intoxication": significantly more positive attitudes toward drunkenness were expressed in the non-exposed cities post-campaign compared with pre-campaign.  "Alcohol as harmless" (response to statements about alcohol's effect on fitness and its use to quench thirst): No significant changes over time within the exposed or non-exposed cities.  "Use of alcohol when entertaining" (items included "A good host makes sure no one's glass is ever empty" and "Non-drinkers are in the minority, so they can't expect non-alcoholic drinks to be available everywhere": no significant changes over time within the exposed or non-exposed cities.  No significant effect of treatment on components which dealt with drinking-driving issues, "drinking to unwind and relieve depression" and "drinking to escape boredom and worries."  Belief that a problem drinker can learn to drink sensibly rather than having to abstain: significant treatment effect with the non-exposed cities shifting toward increased acceptance of the need for abstinence and the media cities shifting toward greater acceptance of the possibility of learning to control problem drinking. | **Exposure**  Across the four intervention groups 60-80% of the young male target group were exposed to about 100 commercial spots. | Attitudes towards alcohol use were affected by the mass media campaign. The campaign inhibited a national trend towards support for greater liberalisation of alcohol. |
| **Dixon (2015)**  Weak quality | **Alcohol consumption**  Changes to recent drinking behaviour did not differ significantly for those drinkers who were aware and not aware of the campaign. | **Intentions**  Significant increase in the proportion of women who drank alcohol indicating they will be likely to reduce their consumption (17% cf 30%; p = 0.026). Personal concern and intentions for drinking did not differ significantly for those drinkers who were aware and not aware of the campaign (p > 0.05).  **Motivation**  Approximately half who drank alcohol and recognised the campaign reported that the advertisement made them feel motivated (either very or somewhat) to reduce their own alcohol consumption.  **Attitudes**  As a result of seeing the ad, I am concerned about the amount of alcohol people in my social circles drink = 80.9%.  **Knowledge**  Significant increase in the proportion of women who knew that drinking alcohol on a regular basis increases cancer risk (p < 0.001), knew of the link between drinking beer and cancer risk (p < 0.001) and were aware that drinking red wine on a regular basis increases cancer risk (p < 0.001). Non-significant increase in proportion who knew the recommended number of standard drinks for low risk in the long term (p = 0.191).  A significantly higher proportion of respondents who were aware of the campaign knew of the links between cancer and alcohol (p < 0.001), wine (p = 0.019) and beer (p < 0.001), and of the drinking guidelines (p = 0.003) compared with those not aware of the campaign. | **Recall**  Unprompted recall of campaign: OR = 1.29, 95% CI 0.80 to 2.08, p = 0.297.  Prompted recognition of campaign: OR = 2.31, 95% CI 1.33 to 4.00, p = 0.003.  Prompted recall of messages (of those who recognised the advertisement):  ‘There is a link between drinking alcohol and getting cancer’ = 95.6%  ‘The more alcohol you drink, and the more often, the greater your risk of cancer’ 81.8%  Recall correct number of drinks (two) mentioned in television advertisement = 94.0%  Prompted recall of the message “You can stay at low risk of developing cancer by drinking no more than two standard drinks on a regular basis” improved significantly among those who recognised the campaign (p = 0.008).  94.0% of those who recognised the campaign recalled the correct number of drinks mentioned in the ad. | The campaign was recognised by the majority of its target audience and achieved its primary objective of increasing awareness of the link between drinking alcohol and cancer among women aged 25–54 years. We also found some evidence to show that knowledge of the NHMRC guidelines for low-risk drinking improved after wave 1 of the campaign. Women who drank more than two standard drinks per day were the most likely to say the ad made them feel more motivated to reduce their drinking. |
| **Grønbæk (2001)**  Weak quality | **Alcohol consumption**  4-5% stated that the campaign had influenced their behaviour, either by making them skip drinks on weekdays or in other ways be more careful. | **Information seeking**  Obtained unit counter:  1992, 1993 and 1994 = 6-7%  Had used or considered using unit counter:  1992, 1993 and 1994 = 2%, representing approximately 80,000 people  **Knowledge**  Knew the sensible drinking limits for their own sex:  Baseline (1990) = 0%  Feb 1993 = 31%  Feb 1997 = 52%  An immediate sharp increase in knowledge of unit guidelines after each October campaign. The figures are lower in the February surveys.  Increase in knowledge of unit measurements for beer and wine since October 1993. | **Awareness**  High awareness in 1990, 1992 and 1993, approximately 80% of the population. The three latest campaigns had lower but still high awareness, 70%. The 1991 campaign had less impact (but evaluation was carried out 5 weeks after the campaign, compared to 1 week in 1990 and 3 weeks from 1992 onwards).  **Attitudinal responses**  Almost no-one regarded the campaigns as unnecessary interference in their private life. 1% expressed annoyance at the campaigns. Approximately 90% thought the campaigns were a good or very good initiative. | Repeating the message of sensible drinking limits has a strong impact on the level of knowledge in the population. |
| **Kaariainen (2008)**  Weak quality | None. | None. | **Onward transmission**  Discussed the pamphlet with other people = 45.7%  **Interaction**  Calculated scores according to the pamphlet (of those who consumed alcohol) = 49.4%  **Awareness**  Noticed the pamphlet = 34.6% | As part of a wide community action, home delivered self-help material is often noticed especially by heavy drinkers. |
| **Karlsson (2005)**  Weak quality | **Alcohol consumption**  No statistically significant changes between the different measurement times, within or between groups.  **Cost** Total cost of designing, printing and distributing the pamphlet = about EUR 8,000. | None | **Recall**  Recalled having received the pamphlet = 26% | Although the results were encouraging, the evidence that self-change pamphlets by themselves had an effect on people’s drinking remains weak. |
| **Plant (1979)**  Weak quality | **Alcohol consumption**  Viewers significantly more likely than non-viewers to report that they sometimes drank alcohol (p < 0.001).  The average alcohol consumption per drinker during the previous week was 14.8 units for viewers and 13.5 units for non-viewers, not significantly different.  33/743 respondents who had seen either the T.V. or newspaper material reported having subsequently reduced their alcohol consumption. | **Treatment seeking**  Increases in yearly referrals to alcoholism treatment agencies.  **Beliefs**  Viewers were more likely than non-viewers to report that problem drinkers could at least sometimes be helped (p < 0.001).  **Knowledge**  Viewers were significantly more likely than non-viewers to be able to name people or agencies offering help to problem drinkers (p < 0.001) or from whom they would advise a problem drinker to seek help (p < 0.01).  Viewers were significantly more likely than non-viewers to be able to name symptoms such as heavy drinking, morning shakes, drunkenness and depression (p < 0.001). | **Exposure**  31.1% reported having seen at least one of the newspaper advertisements.  659 reported having seen at least one of the T.V. films.  About a quarter reported that they had seen neither the T.V. films nor the newspaper advertisements.  Nearly twice as many respondents reported having seen the T.V. films as reported having seen the newspaper advertisements.  **Recall**  87% of viewers named at least one feature of the T.V. films.  Average number of TV film features viewers were able to list = 2.5 items in one region, 2.0 items in another region. | The main impact of the campaign, as judged by the surveys, was marginally to increase factual knowledge. The results do not support the view that the T.V. films prompted viewers to drink less. |
| **Siriwardhana (2013)**  Weak quality | None. | None. | **Recall**  Posters = 75%.  Leaflets = 43%.  **Attitudinal responses**  Approval of both posters and leaflets was <15%.  **Interaction**  Watched DVD of the street dramas = 52%. | A community-based education program had high acceptance. |
| **Wallack (1982)**  Moderate quality | **Alcohol consumption**  Distributions of variables measuring quantity/frequency of drinking remained unchanged over time. The proportion of high-maximum drinkers, those drinking five or more per occasion, was relatively constant for both adults and youth.  Both youth and adult data showed no differences in the frequency with which they reported drinking to intoxication when baseline and final surveys were compared. | **Intention**  Some respondents indicated they might change their behaviour but little evidence was found to substantiate this claim.  **Knowledge**  The initial survey revealed that respondents were already quite well informed about many of the items - and this did not change markedly during the demonstration.  For intervention youth, slightly more than 20% indicated they had received new information as a result of the “Winners” program. This had changed little from the interim survey.  **Attitudes**  No change in the expected direction was found on any of the following items:  I keep in mind how I’m going to get home safely when I’m drinking; I know my limit and usually stick to it; I sometimes drive when I’m drunk enough to be in trouble if stopped by the police. Respondents seemed to remain consistent over time in their concern about how much alcohol they consume and the possible negative effects of such consumption. | **Understanding**  A substantial increase from the interim to the final survey in the % of intervention respondents who were able to correctly interpret the campaign slogan.  Approximately 4% of control respondents correctly interpreted at least one of the “Winners” commercials.  **Recall**  76% intervention and 39% control claimed to have seen at least one of the commercials. Over 90 percent of intervention youth recognized at least one television spot. No commercial was recognised by more than 14% of control respondents.  **Awareness**  Awareness of alcohol-related messages in the environment from sources used by the demonstration remained constant in the intervention area and declined for the control area. Baseline data were similar for the two sites. Awareness of alcohol messages from non-program sources (newspapers and magazines) declined in both the intervention and control areas.  **Onward transmission/discussion**  On a measure of interpersonal contacts (discussions concerning alcohol problems with people in respondent’s family or household, or with people at work or school), control adults reported some increased frequency, but respondents in the control site showed an increase of similar magnitude.  The youth data provide a picture generally similar to that of the adults. Relative awareness of messages from program sources was increased in the intervention area. Awareness from non-program sources declined over time for both sites. Intervention youth reported about the same frequency of interpersonal contacts at the conclusion of the demonstration as they did on the baseline. Control respondents, however, were more likely to report having had interpersonal contacts “fairly often.” | Overall it appeared that on variables related to the intermediate objectives of recognition, recall, and comprehension, the demonstration was successful. Although widespread recognition and recall of demonstration messages was clearly achieved, success in obtaining desired outcome goals was modest. |
| **Campaigns targeting young people and/or their parents** | | | | |
| **Atkinson (2011)**  Weak quality | None. | None. | **Interaction**  1313 online comments in response to the episodes on the E4 and YouTube websites. Limited audience conversation relating to alcohol and alcohol-related content.  **Identification**  A lack of awareness that the episodes were designed as an alcohol awareness campaign. In contrast, viewers seemed to reject the depictions of alcohol portrayed, and identified with, or admired, the central characters.  **Attitudinal responses**  The E4 website asked viewers to express their views on the series by rating each episode as either ‘Yay’ or ‘Nay’. Based on ratings by 14558 viewers, the rating of episodes as ‘Yay’ ranged from 44% to 90%. | New media provides creative new opportunities to engage young people with health-promoting messages. However, although new ways of delivery are important they should be part of a co-ordinated and internally consistent campaign, present realistic depictions of alcohol use, and be based upon clear evidence-based principles. |
| **Flynn (2006)**  Strong quality | **Alcohol consumption**  No significant difference in beer drinking within last 30 days between the exposed and comparison groups. Similar changes in both groups. | **Norms & self-efficacy**  No effect on psychosocial mediators of alcohol use. | **Attitudinal responses**  Of the youth who saw the television messages:  Liked them = 70%  Liked them a lot = 35%  Of those who heard the radio messages:  Liked them = 75%  Liked them a lot = 35%  Of the parents who recognised any message:  Messages were helpful = 96%  Messages were very helpful = 41%  **Recall**  *Main post-campaign survey:*  Mean messages seen/heard by students in exposed group = 7.8, of which 3.6 were television and 4.2 were radio messages.  Seen/heard none of the messages = 18.7%.  Messages seen/heard at least once by >50% participants = 3 of 8 television and 1 of 11 radio messages.  *Parent survey:*  Recognised any of the 6 messages = 35%.  **Exposure**  Change in reported level of exposure to alcohol prevention messages from multiple sources:  Significant increase in reported exposure via television (p = 0.03) and radio (p < 0.01) in the exposed versus the non-exposed area; no significant differences in exposure via parents, school classes, friends or magazines/newspapers. | The media interventions did not significantly affect alcohol use or its mediators. |
| **Kelley (2000)**  Weak quality | None. | **Self-efficacy**  Grades 10-12: Increase in self-efficacy (what respondents can do to stop the use of a substance) by year for alcohol (p = 0.02).  Grades 8-9: Univariate analyses indicated an increase for all communities in what respondents can do to stop the use of alcohol (p = 0.05).  **Community environment**  Grades 10-12: Girls in one community reported more being done to stop the use of alcohol.  Grades 8-9: Significant differences in community environment by year for alcohol (p = 0.05) and by the interaction between year and community for alcohol (p = 0.004).  Significant increase in all communities in how much has been learned from the media about the dangers of smoking and alcohol use.  **School environment**  Two communities showed significant increases in how much has been learned about the dangers of tobacco and alcohol use from school programs. The remaining two communities showed significant decreases in how much was learned about alcohol use from school programs.  Grades 8-9: A significant change was found in school environment for alcohol (p = 0.01).  In comparing means, a significant decrease in what teachers do to stop alcohol use was found for all communities. | None. | Mixed results. Several meaningful changes were found when comparing the baseline and follow-up responses. |
| **Kypri (2005)**  Moderate quality | **Alcohol consumption (unsupervised drinking)**  Teenagers in the intervention areas had lower odds of being supplied alcohol for unsupervised drinking but this result was not statistically significant (OR = 0.73; 95% CI: 0.43, 1.25).  **Alcohol consumption (binge drinking)**  Teenagers in the intervention areas had higher odds of binge drinking at follow-up but this result was not statistically significant (OR =1.28; 95% CI: 0.65, 2.50). | None. | **Onward transmission/discussion**  28% of parents in the intervention districts said they discussed issues surrounding unsupervised drinking more with their teenager during the campaign than before it commenced, while 20% said they discussed unsupervised drinking more frequently with other adults during this time. Of the parents who answered ‘more often’ to the question concerning discussion with their teenagers, 76% attributed this to the campaign. Of the parents who answered ‘more often’ to the question concerning discussion with other parents, 74% attributed this to the campaign.  **Recall**  Statistically significantly more campaign items reported seen by Waitaki (intervention) than Clutha (control) residents (p < 0.01).  The most commonly seen campaign items were newspaper advertisements (65%), newspaper articles and photos (62%), stickers in bottle stores (52%), radio advertisements (41%) and billboards (37%). | Unsupervised drinking might have increased in the intervention districts had it not been for the campaign. |
| **Scheier (2010)**  Strong quality | **Alcohol consumption**  Alcohol use increased from the younger age to the older age. In the older age group, increase in awareness was associated with decrease in binge drinking. | None. | **Awareness**  Campaign awareness increased with age from 12 to 14 years apart from television watching and then declined in older ages. Awareness in terms of radio listening and recalling of stories was associated with faster rates of increase of binge drinking in younger age group (p < 0.05). | When they were younger, these youth accelerated their drug use and reported increasing amounts of campaign awareness. When they were older, increasing awareness was associated with declines in binge drinking and cigarette smoking. |
| **Surkan (2003)**  Weak quality | None. | None. | **Onward transmission/discussion**  71.6% had discussed the consequences of alcohol one or more times with at least one of their children 10–17 years old in the past 30 days.  The number of discussions in the past 30 days that parents or guardians reported having with their children 10–17 years old about alcohol was associated with unassisted recall of the radio spot (p < 0.02).  Respondents who recalled the spot were more likely to report having three or more discussions with their children.  **Recall**  Had heard advertising on the radio during the past 30 days about ‘‘people under 21 and consequences of alcohol use’’ = 32.6%.  When asked what the radio advertising said, judged to have described the content of the Massachusetts Department of Public Health’s radio advertisement (‘‘unassisted recall’’) = 12.4% | The findings suggest the potential benefit of paid media campaigns designed to encourage parents to talk with their children about alcohol. |
| **Trees (2015)**  Weak quality | **Change in alcohol use**  Aware of someone changing his/her alcohol use because of the campaign:  About one-quarter of interviewees who answered the question (about 15% of all interviewees).  The general finding was that the very limited exposure to the radio advertisements in remote communities was likely to have had hardly any impact on alcohol use. | None. | **Onward transmission**  About half the interviewees who responded to the relevant question had heard others discussing the television advertisements but fewer heard comments about the radio advertisements, indicating that they had not aroused a high level of public debate.  **Attitudinal/emotional responses**  Participants were not very interested in the campaign. The survey interviewees considered the television advertisements to be of high quality. Almost all interviewees said that excessive drinking was a big community problem and a number of participants suggested that the campaign was a very good idea. Some others criticised the way the campaign was carried out and suggested alternative approaches.  About two-thirds of interviewees who answered the question thought that the television community announcements (i.e. advertisements) were interesting and effective for the target audience. Half the people who responded to this question agreed that the advertisements were effective for them. Some commented that advertisements should be targeted at older people, whose drinking behaviour has a strong impact on families, rather than at young people like themselves.  Quality of the radio campaign:  Those who responded to this question were only slightly more likely (29% cf. 23%) to say that it was effective rather than ineffective.  'Do you think the Radio ads made the message relevant to young people?':  • for the full sample, those who had heard the advertisements were somewhat more likely to say that they were effective than non-effective for young people  • young Indigenous interviewees were less likely to say the advertisements were effective, with almost twice as many saying 'no' as 'yes'. However, disengagement of youth was not universal.  **Credibility**  The focus group results indicated that the key feature of earlier positive responses to two Goolarri advertisements, 'Boys rap' and 'Girls rap', was involvement of young people in the design and production of the advertisements.  **Awareness**  Reasonable awareness of the television campaign (about 63%) and a somewhat lower response rate to the radio advertisements (49%).  **Recall**  About half of the interviewees remembered at least one slogan and one theme. The radio advertisements were somewhat less effective in this regard.  **Exposure**  Very few residents in communities remote from Broome were exposed to the radio advertisements. | Two of the most interesting findings from the consultancy study: disengagement of youth and the rise of mobile media use. Youth involvement in creating, accessing and sharing knowledge facilitates health promotion. |
| **van Gemert (2011)**  Weak quality | None. | None. | **Recall**  Recognised the message of the Drinking Nightmare Campaign = 74.7%. | Whilst a high proportion of the target group recognised the campaign, reported frequent RSOD had statistically significantly lower odds of recognising the campaign message. |
| **van Leeuwen (2013)**  Weak quality | **Alcohol consumption**  Model 1 including viewing status as predictor was not able to predict changes in the quantity of alcohol consumed from pre- to post-test (p = 0.37). In model 2 viewing status was a significant predictor of changes from pre- to post-test in the quantity of alcohol consumed (p < 0.05).  Non-viewers increased, whereas viewers slightly decreased, their number of alcoholic consumptions per occasion from pre- to post-test. | **Intentions**  Viewing status significantly predicts changes in intentions to decrease alcohol use from pre- to post-test (p < 0.01).  **Beliefs**  The models with viewing status, education level, or an interaction of these variables as predictors were not able to predict changes in positive or negative outcome expectancies.  **Perceived norms**  Change in perceived normative pressure between pre- and post-test is significantly predicted by viewing status (p < 0.01). The results indicate an increase in normative pressure among viewers and a decrease among non-viewers. | None. | Viewers perceived the narratives to be credible and enjoyable but did not relate to the characters in the narratives. However, exposure to the intervention predicted desired changes in alcohol drinking behaviour, intentions to decrease alcohol use, and perceived normative pressure, especially in less educated viewers. |
| **Campaigns targeting pregnant women or women of childbearing age** | | | | |
| **Awopetu (2008)**  Historically controlled study | None. | **Information seeking**  49 FAS-related telephone calls were received by the Family Health Line, compared to 5-6 FAS related telephone calls received in a similar period in 2005-2006. | None. | The intervention reached a wide geographic audience, provoking interest beyond the initial target area and expected age ranges. The results, however, point to the difficulty of raising awareness of FASD. |
| **Casiro (1994)**  Weak quality | None. | **Knowledge**  A significantly higher proportion of respondents after the public awareness campaign thought that drinking alcohol during pregnancy could cause mental (p < 0.001), physical (p < 0.001) and behavioural (p < 0.001) abnormalities in the baby.  Most women surveyed either before or after the public awareness campaign knew that one alcoholic drink per day during pregnancy could place the baby at risk (p = 0.334).  The pre-campaign perception of risk to the baby for smaller amounts of alcohol was markedly lower, showing a significant improvement after the public awareness campaign. For drinking alcohol once a week 68% of women before the campaign and 74.2% after the campaign (p < 0.001) believed such consumption would put the baby at risk, while 50% of women before the campaign and 59.8% after the campaign (p < 0.001) indicated that drinking alcohol once a month would place the baby at risk. | **Awareness**  Statistically significant change in source of information for "television" before and after the campaign (p < 0.001). | A television campaign highlighting the potential risks of alcohol consumption during pregnancy increased the knowledge level of women of childbearing age attending a variety of physicians' offices and community-based clinics located throughout Manitoba. Significant differences were only found in the answers to alcohol-related questions and the proportion of respondents who attributed the information to television increased significantly after the campaign. |
| **Hanson (2012)**  Weak quality | **Alcohol consumption**  Agreed the campaign had caused them to reduce their drinking = 71.8%. | **Knowledge**  Agreed it increased their knowledge on FAS = 91.6%.  Agreed it increased their knowledge on effect of alcohol consumption during pregnancy = 93.3%. | **Identification**  Campaign culturally appropriate = 85.7%. | The campaign was seen as culturally appropriate, increased knowledge, and decreased actual drinking. |
| **Lowe (2010)**  Moderate quality | None. | **Knowledge**  Mean scores improved in the intervention group and stayed the same in the control group. The difference in the change between groups was statistically significant, t(698) = 2.120, p < 0.05. | **Onward transmission/discussion**  Watched the videotape/DVD with at least one other person = 39.7% of those who watched it.  Discussed the videotape/DVD with another person = 54.5% of those who watched it.  Passed on specific information from the videotape/DVD to other women = 50.0% of those who watched it.  Passed on their copy of the videotape/DVD = 47.4% of those who watched it.  Significantly more women in the intervention group (58.3%) had talked to friends about alcohol use during pregnancy, compared with 49.4% of controls (p = 0.05.)  **Interaction**  Watched the videotape/DVD = 62.2%.  **Recall**  Recalled seeing the commercial (both groups were exposed):  Intervention group = 64.1%, Control group = 48.5%, (p < 0.001).  Recalled receiving the videotape/DVD (only intervention group exposed) = 100%. | This study revealed a modest increase in social network communication about the use of alcohol during pregnancy among the pregnant women who were given exposure to the multimedia intervention of television commercial, videotape/  DVD, and written pamphlet.  The intervention increased knowledge about several aspects of the consequences of alcohol use in pregnant women. |
